# The West Virginia Dental Law

**Published:** 1881-05

**Authors:** 


### ARTICLE IX.

### *The West Virginia Dental Law.*

A Bill to regulate the practice of dentistry in West Virginia; and to protect the people against empiricism in relation thereto.

Be it enacted by the Legislature of West Virginia:

Section 1. It shall be unlawful for any person to engage in the practice of dentistry for compensation in this State, unless such person shall have received a diploma from some Dental College, duly incorporated under the laws of this State or some one of the United States, or foreign government, in which is annually delivered in good faith a full course of lectures and instruction in dentistry, or shall have obtained a license from a Board of Dentists, duly authorized by the authorities of this or some one of the United States, in the manner hereinafter mentioned.

Section 2. It shall be the duty of the Board of Public Works to appoint nine dentists, learned in the profession, three of whom shall be appointed in each Congressional district, who shall constitute a board for the examination of applicants in their own districts, and before which all applicants for license to practice dentistry shall appear and be examined touching his proficiency in said art or profession, and if two or more of said board shall deem the said applicant qualified to practice said profession, they shall sign said license, for making which examination the said examiners shall have a fee of two dollars each, to be paid by the applicant; provided, that nothing in this act shall prevent any person from extracting teeth or in any manner interfere with any person now engaged in the practice of dentistry in this State. The term of office of such board shall be five years.

Section 3. Any person violating the provisions of this act shall be deemed guilty of a misdemeanor, and on conviction thereof shall be fined not less than ten or more than one hundred dollars.

---

